# Supplementary material for: Exploring breast cancer awareness and screening practices amongst rural women in The Gambia: Community-based cross-sectional study
Source: PLoS One. 2025 May 12;20(5):e0307785. doi: 10.1371/journal.pone.0307785 (PMC12068578; doi:10.1371/journal.pone.0307785)

PLOS ONE

Exploring Breast Cancer Awareness and Screening Practices Amongst Gambian Women in the Gambia; Community-based- cross-sectional study

--Manuscript Draft--

Manuscript Number:	PONE-D-24-19264
Article Type:	Research Article
Full Title:	Exploring Breast Cancer Awareness and Screening Practices Amongst Gambian Women in the Gambia; Community-based- cross-sectional study
Short Title:	Breast Cancer Screening Practices among Gambian Women
Corresponding Author:	Bakary Kinteh<br>Gambia College<br>Banjul, GAMBIA
Keywords:	breast cancer; Screening Practices; Rural Gambian Women
Abstract:	Background<br><br>Breast cancer prevalence is on a constant rise, and it remains the second most diagnosed cancer among women in The Gambia. More than 15% of Gambian women were diagnosed with breast cancer and half of those diagnosed died of the disease in 2020. Thus, we explored the breast cancer awareness and screening practices of rural Gambian women.<br><br>Methods<br><br>A community-based cross-sectional study was conducted among Northern Gambian rural women. We recruited 985 rural women from two local government authorities using a multi-stage sample technique and Kobo toolbox was used for interviews with a structured questionnaire adapted from a study among female university students. Data was analyzed using SPSS version 28; continuous variables were presented in frequencies and percentages while categorical variables were illustrated using charts. Pearson chi-square test was conducted to determine the correlation and the Multivariable logistic regression test was to determine the factors associated with breast cancer screening practices declaring statistically significant at p-valve <0.05.<br><br>Results<br><br>The mean age was 32 (±12.6) years and 34% of our respondents were aged 18—24 years. Majority (87.7%) were aware of breast cancer and mass media was their source of information. Obesity/Overweight (35.2%), family history of breast cancer (29.0%) and old age (16.6%) were identified as risk factors. However, less than 13% ever practiced breast cancer screening and among them, 62.6% performed clinical breast examination. The significant predictors of breast cancer screening practices were Civil servant (aOR =2.778, 95% CI =1.174 – 6.573), Student (aOR = 3.111, 95% CI =1.453 – 6.663), Parity two (aOR = 0.061, 95% CI = 0.005 – 0.791), parity three (aOR = 0.075, 95% CI = 0.006 – 0.967), and parity five (aOR =0.065, 95% CI = 0.005 – 0.877).<br><br>Conclusion<br><br>The awareness of breast cancer among rural women was above the average in this study. However, their screening practices were not optimal and their reasons for not screening was centred on lack of knowledge, service unavailability and high cost. We implore on MoH and her partners to ensure breast cancer screening services available, accessible, and affordable to rural women.
Order of Authors:	Bakary Kinteh
	Fatoumatta Jitteh
	Mansour Badjie

Powered by Editorial Manager® and ProduXion Manager® from Aries Systems Corporation

	Amadou Barrow
	Lamin Jaiteh
**Additional Information:**	
**Question**	**Response**
**Financial Disclosure**<br><br>Enter a financial disclosure statement that describes the sources of funding for the work included in this submission. Review the submission guidelines for detailed requirements. View published research articles from *PLOS ONE* for specific examples.<br><br>This statement is required for submission and **will appear in the published article** if the submission is accepted. Please make sure it is accurate.<br><br>**Funded studies**<br>Enter a statement with the following details:<br>• Initials of the authors who received each award<br>• Grant numbers awarded to each author<br>• The full name of each funder<br>• URL of each funder website<br>• Did the sponsors or funders play any role in the study design, data collection and analysis, decision to publish, or preparation of the manuscript?<br><br>Did you receive funding for this work?	The author(s) received no specific funding for this work.
**Competing Interests**<br><br>Use the instructions below to enter a competing interest statement for this submission. On behalf of all authors, disclose any competing interests that could be perceived to bias this work—acknowledging all financial support and any other relevant financial or non-financial competing interests.<br><br>This statement is required for submission and **will appear in the published article** if	The authors declare and affirm that they do not have any personal, financial, or professional interests that could potentially influence or tamper with the validity of the results. This work is therefore free of biases that could compromise its scientific integrity.

the submission is accepted. Please make sure it is accurate and that any funding sources listed in your Funding Information later in the submission form are also declared in your Financial Disclosure statement.<br><br>View published research articles from *PLOS ONE* for specific examples.<br><br>**NO authors have competing interests**<br><br>Enter: *The authors have declared that no competing interests exist.*<br><br>**Authors with competing interests**<br><br>Enter competing interest details beginning with this statement:<br><br>*I have read the journal's policy and the authors of this manuscript have the following competing interests: [insert competing interests here]*<br><br>* typeset	
**Ethics Statement**<br><br>Enter an ethics statement for this submission. This statement is required if the study involved:<br><br>• Human participants<br>• Human specimens or tissue<br>• Vertebrate animals or cephalopods<br>• Vertebrate embryos or tissues<br>• Field research<br><br>Write "N/A" if the submission does not require an ethics statement.<br><br>General guidance is provided below. Consult the submission guidelines for detailed instructions. **Make sure that all information entered here is included in the Methods section of the manuscript.**	Ethical approval for this study was received from the Research Ethics Committee (REC) of Edward Francis Small Teaching Hospital (EFSTH_REC_202-038. Permission to conduct the study within the communities was verbally obtained from community entry points such as the Alkolos and Village Development Committees. Voluntary Informed consent was obtained from each study participant verbally or through a signed consent form where participants can read or write before the onset of the interview and maintained throughout the data collection process.

Powered by Editorial Manager® and ProduXion Manager® from Aries Systems Corporation

**Format for specific study types**<br><br>**Human Subject Research (involving human participants and/or tissue)**<br>• Give the name of the institutional review board or ethics committee that approved the study<br>• Include the approval number and/or a statement indicating approval of this research<br>• Indicate the form of consent obtained (written/oral) or the reason that consent was not obtained (e.g. the data were analyzed anonymously)<br><br>**Animal Research (involving vertebrate animals, embryos or tissues)**<br>• Provide the name of the Institutional Animal Care and Use Committee (IACUC) or other relevant ethics board that reviewed the study protocol, and indicate whether they approved this research or granted a formal waiver of ethical approval<br>• Include an approval number if one was obtained<br>• If the study involved *non-human primates*, add *additional details* about animal welfare and steps taken to ameliorate suffering<br>• If anesthesia, euthanasia, or any kind of animal sacrifice is part of the study, include briefly which substances and/or methods were applied<br><br>**Field Research**<br><br>Include the following details if this study involves the collection of plant, animal, or other materials from a natural setting:<br>• Field permit number<br>• Name of the institution or relevant body that granted permission	
**Data Availability**<br><br>Authors are required to make all data underlying the findings described fully available, without restriction, and from the time of publication. PLOS allows rare exceptions to address legal and ethical concerns. See the PLOS Data Policy and FAQ for detailed information.	Yes - all data are fully available without restriction

Powered by Editorial Manager® and ProduXion Manager® from Aries Systems Corporation

A Data Availability Statement describing where the data can be found is required at submission. Your answers to this question constitute the Data Availability Statement and **will be published in the article**, if accepted.<br><br>**Important:** Stating 'data available on request from the author' is not sufficient. If your data are only available upon request, select 'No' for the first question and explain your exceptional situation in the text box.<br><br>Do the authors confirm that all data underlying the findings described in their manuscript are fully available without restriction?	
**Describe where the data may be found in full sentences. If you are copying our sample text, replace any instances of XXX with the appropriate details.**<br><br>• If the data are **held or will be held in a public repository**, include URLs, accession numbers or DOIs. If this information will only be available after acceptance, indicate this by ticking the box below. For example: *All XXX files are available from the XXX database (accession number(s) XXX, XXX.).*<br>• If the data are all contained **within the manuscript and/or Supporting Information files**, enter the following: *All relevant data are within the manuscript and its Supporting Information files.*<br>• If neither of these applies but you are able to provide **details of access elsewhere**, with or without limitations, please do so. For example:<br><br>*Data cannot be shared publicly because of [XXX]. Data are available from the XXX Institutional Data Access / Ethics Committee (contact via XXX) for researchers who meet the criteria for access to confidential data.*<br><br>*The data underlying the results presented in the study are available from (include the name of the third party*	The data used to support the findings of this study are available from the Corresponding author upon reasonable request at: bakarykinteh461@gmail.com

and contact information or URL).<br>• This text is appropriate if the data are owned by a third party and authors do not have permission to share the data.<br><br>* typeset	
Additional data availability information:	Tick here if the URLs/accession numbers/DOIs will be available only after acceptance of the manuscript for publication so that we can ensure their inclusion before publication.

Powered by Editorial Manager® and ProduXion Manager® from Aries Systems Corporation

Manuscript
Click here to access/download;Manuscript;PLOS_ONE_Final_Manuscript_B

Exploring Breast Cancer Awareness and Screening Practices Amongst Women in The Gambia; Community-based- cross-sectional study

Bakary Kinteh[1,2*], Fatoumatta Jitteh[2,3], Mansour Badjie[1], Amadou Barrow [1,4,5], Lamin Jaiteh[2,3,6]

1. School of Public Health, Gambia College
2. Solace Foundation, The Gambia
3. Edward Francis Small Teaching Hospital, Banjul The Gambia
4. Department of Public & Environmental Health, School of Medicine & Allied Health Sciences, University of The Gambia
5. Department of Epidemiology, University of Florida, United States of America
6. School of Medicine and Allied Health Sciences, University of The Gambia

Correspondence:

Mr. Bakary Kinteh
E-mail: bakarykinteh461@gmail.com; Phone: +220 3597841

Abstract

Background: Breast cancer prevalence is on a constant rise, and it remains the second most diagnosed cancer among women in The Gambia. More than 15% of Gambian women were diagnosed with breast cancer and half of those diagnosed died of the disease in 2020. Thus, we explored the breast cancer awareness and screening practices of rural Gambian women.

Methods: A community-based cross-sectional study was conducted among Northern Gambian rural women. We recruited 985 rural women from two local government authorities using a multi-stage sample technique and Kobo toolbox was used for interviews with a structured questionnaire adapted from a study among female university students. Data was analyzed using SPSS version 28; continuous variables were presented in frequencies and percentages while categorical variables were illustrated using charts. Pearson chi-square test was conducted to determine the correlation and the Multivariable logistic regression test was to determine the factors associated with breast cancer screening practices declaring statistically significant at p-valve <0.05.

Results The mean age was [illegible]2 (±12.6) years and 34% of our respondents were aged 18—24 years. Majority (87.7%) were aware of breast cancer and mass media was their source of information. Obesity/Overweight (35.2%), family history of breast cancer (29.0%) and old age (16.6%) were identified as risk factors. However, less than 13% ever practiced breast cancer screening and among them, 62.6% performed clinical breast examination. The significant predictors of breast cancer screening practices were Civil servant (aOR =2.778, 95% CI =1.174 – 6.573), Student (aOR = 3.111, 95% CI =1.453 – 6.663), Parity two (aOR = 0.061, 95% CI = 0.005 – 0.791), parity three (aOR = 0.075, 95% CI = 0.006 – 0.967), and parity five (aOR =0.065, 95% CI = 0.005 – 0.877).

Conclusion: The awareness of breast cancer among rural women was above the average in this study. However, their screening practices were not optimal and their reasons for not

screening was centred on lack of knowledge, service unavailability and high cost. We implore on MoH and her partners to ensure breast cancer screening services available, accessible, and affordable to rural women.

Key words: Breast Cancer, Screening Practices, Rural Gambian Women

Introduction

Globally, Breast cancer remains one of the most commonly diagnosed cancers among women, it is a leading cause of cancer-related deaths in women, accounting for 23% of cancer cases and 14% of deaths worldwide(Arnold et al., 2022). The high morbidity and mortality rates do not exempt low and middle countries where the incidence of breast cancer has been increasing rapidly largely attributed to lifestyle changes, alterations in reproductive health factors, and longer life expectancy (Pal et al., 2021). According to recent World Health Organization (WHO) reports, in 2022, 157 of 185 countries registered breast cancer as the most prevalent cancer affecting women (WHO, 2024). In The Gambia breast cancer is the second most diagnosed cancer second only to cervical cancer. The incidence of the disease is gradually increasing among women. More than 50% of diagnosed patients died of the disease in 2020. Breast cancer prevalence in The Gambia was estimated at 11.25 in every100, 000 women in the population (Globocan, 2021).

A Cancer Registry Unit under the Ministry of Health was tasked with the country's cancer data from diagnosis to treatment. However, the country is limited in its capacity to meet the initiative set by the WHO to contribute significantly to saving 3 million lives from breast cancer mortality by 2040 (WHO, 2024). Limitations in capacity include breast cancer diagnosis, treatment, and patient management. The ministry is contending with difficulties in ensuring early detection and timely breast cancer diagnosis, as only 50% (52/102) of healthcare facilities can provide a clinical diagnosis of breast cancer. In The Gambia, the breast cancer burden is exacerbated by the fact that women have to travel up to 45 km to access healthcare facilities (Sanyang et al. 2021). Late breast cancer diagnosis leads to poor prognosis of the disease among women and The Gambia just like many other Sub-Saharan African countries is challenged in this regard.

Evidence suggests that it takes a woman on average 3- 6 months to seek the attention of a healthcare worker for her breast symptoms (Espina et al., 2017). This delay can make the difference between life and death. It has a significant impact on both prognosis and survival rates among breast cancer patients. The contributory causes of poor prognosis of breast cancer are multifactorial, ranging from a lack of awareness, myths and concepts and socio-economic factors in addition to inadequate access to accurate and modern diagnostic facilities. (Kohler et al., 2017; Pace & Shulman, 2016, 2016).

A majority of breast cancer patients only receive specialist care at advanced stages of the disease. A lack of knowledge about breast cancer among women in the country was highlighted in a study conducted by Kinteh et al., which focused on female university students (Kinteh et al., 2023). The study revealed that female university students had good knowledge about breast cancer; however, breast cancer screening practises were still poor, as up to 75% of the respondents had never had any form of breast cancer screening (Kinteh et al., 2023).

For women less than 70 years old, The WHO has a target of reducing mortalities to 25% by 2030 and up to 40% by 2040 through the Global Breast Cancer Initiative (GBCI). To achieve this target, 3 key strategies have been identified: early detection through effective health promotion strategies, timely and accurate diagnosis, and prompt treatment (WHO, 2024).

In The Gambia, the Health Policy of the Ministry of Health 2021–2023 has underscored breast cancer prevalence as the second most prevalent cancer in the country among non-communicable diseases (Ministry of Health, 2021). However, strategies for addressing breast cancer are limited. Furthermore, the country faces significant limitations in terms of the effective implementation of the three GBCI strategies.

Thus, the aim of this study was to explore breast cancer awareness and screening practices of rural Gambian women. The study findings will therefore assist the Ministry of Health in designing and implementing strategies to combat breast cancer in The Gambia.

Material and Methods

Study design and setting

A community-based cross-sectional study design was used to assess breast cancer awareness and screening practices in women of reproductive age residing along the Northern Strip of The Gambia. The Gambia is divided into the Northern and Southern parts by the river Gambia. The country's northern half comprises two local government authorities: the Kerewan and Kuntaur Area Councils. The Kerewan Area Council has a total population of 225,516 inhabitants and the Kuntaur Area Council has a total population of 98,966 (GBoS, 2013).

Study population

Women aged ≥ 18 years were recruited from various communities to participate in this study. Only women residing in the communities for at least 12 months were included in this study. Guests and non-resident women were therefore excluded from this study to ensure conformity and neutrality.

Sample size determination

Epi info version 7.0 was used to determine the sample size of the study. A combined population of the two local administrative authorities was used as the base population, and a confidence interval of 99% with 50% precision was used to calculate the minimum sample size. A total of 985 women were enrolled in our study.

Sampling strategy

The study participants were recruited through a multistage sampling strategy. At first, the study area was clustered into two sites using the local government administrative authority designation (the Kerewan Area Council and Kuntaur Area Council). This was followed by a proportionate-to-size sampling technique to select districts (four districts in Kerewan and three districts in Kuntaur). Simple random sampling was used to select five communities from each district. Subsequently, another proportionate-to-size sampling technique was used to select eligible households in each community. Ultimately, a systematic sampling technique was used to select households in the community, and simple random sampling was used to select participants in households with more than a single eligible respondent.

Data collection and management

A closed-ended structured questionnaire was used in this study. The questionnaire was adapted from a similar study done among female university students in The Gambia. The questionnaire was adjusted and standardized to a community-level study, with variables comprising a sociodemographic section, breast cancer awareness section, and screening practices section among women at the community level. The sociodemographic section of the questionnaire contained variables such as the age of the respondents in years, ethnicity, religion, nationality, level of education, current occupation, marital status, and parity of women. Breast cancer screening practices were assessed using four questions: breast cancer screening practises, screening method (breast self-examination, clinical breast examination, and mammography), main push factors for screening and main reasons hindering breast cancer screening.

The questionnaire was developed using an online data collection application known as the Kobo Toolbox. Data were collected in the field using this online platform. The data collectors were students from the School of Medicine and Allied Health Sciences, University of The Gambia Medical Students, and the School of Public Health, Gambia College. The data collectors received a day-long training on Kobo Toolbox, interview techniques, and translation of the English version to three other local languages namely, Mandinka, Wolof, and Fula. The study was piloted among women in West Coast Region before proceeding to the field to ensure reliability and consistency of the questions. The data collection at the communities was carried out by over 150 research assistants from Solace Foundation and School of Public Gambia College from the 24th to 31st January 2024.

The online data collection method was monitored by team supervisors in the field to ensure and maintain data accuracy. The data were synchronized to a server that also monitored for minor errors and then downloaded from the Kobo toolbox to Microsoft Excel, where further error checks were performed and corrected.

Statistical analysis

The cleaned data was finally imported to the IBM Statistical Package for Social Science (SPSS) version 28 for analysis. The Continuous variables were presented in means and standard deviations, while the categorical variables were represented in both frequencies and percentages. Pearson Chi-square test was used to test for association between outcome and covariates while multivariate logistic regression was used to determine predictors of breast cancer screening practices. The statistical significance was set at $p < 0.05$ with 95% confidence interval

Ethical considerations

Ethical approval for this study was received from the Research Ethics Committee (REC) of Edward Francis Small Teaching Hospital (EFSTH_REC_202-038. Permission to conduct the study within the communities was verbally obtained from community entry points such as the Alkolos and Village Development Committees. Voluntary Informed consent was obtained from each study participant verbally or through a signed consent form where participants can read or write before the onset of the interview and maintained throughout the data collection process.

Results

As shown in Table 1, 1012 women were recruited from thirty-five communities, with a response rate of 97.3% (985/1012). The mean age of the respondents was 32 (±12.6) years and 34% of the respondents were aged 18–24 years. Mandinka ethnicity accounted for 39%, 99.5% practiced Islamic religion, 2.3% were non-Gambian, and nearly half (46.2%) had no formal education. Housewives accounted for 30% of the sample, nearly two-third of them were married, and a little above a quarter (26.8%) were nulliparous.

Table 1: showing the socio-demographic characteristics and breast cancer screening practice of the participants

Variable	Practiced breast cancer screening			p-valve
	No(%)	Yes(%)	Total(%)	
**Age of respondents**				0.235
18 – 24	283(27.6)	52(5.3)	335(34.0)	
25 – 31	238(24.2)	25(2.5)	263(26.7)	
32 – 38	125(12.7)	20(2.0)	145(14.7)	
39 – 45	91(9.2)	11(1.1)	102(10.4)	
46 & Above	124(12.6)	16(1.6)	140(14.2)	
**Ethnicity**				0.463
Mandinka	345(35.0)	39(4.0)	384(39.0)	
Fula	129(13.1)	24(2.4)	153(15.3)	
Wolof	323(32.8)	50(5.1)	373(37.9)	
Serahuli	6(0.2)	2(0.2)	8(0.8)	
Jola	37(3.8)	7(0.7)	44(4.5)	
Serer	19(1.9)	2(0.2)	21(2.1)	
Others	2(0.2)	0(0.0)	2(0.2)	
**Religion**				0.821
Islam	857(87.0)	123(12.5)	980(99.5)	
Christianity	4(0.4)	1(0.1)	5(0.5)	
**Nationality**				
Gambian	841(85.4)	121(12.3)	962(97.7)	
Non-Gambian	20(2.0)	3(0.3)	23(2.3)	
**Education level**				**0.006***
No formal education	405(41.1)	50(5.1)	455(46.2)	
Primary education	145(14.7)	19(1.9)	164(16.6)	
Secondary education	228(23.1)	30(3.0)	258(26.2)	
Tertiary education	83(8.4)	25(2.5)	108(11.0)	
**Current Occupation**				**<0.001***
Unemployed	111(11.3)	12(1.2)	123(12.5)	
Civil servant	49(5.0)	14(1.4)	63(6.4)	
Student	96(9.7)	32(3.2)	128(13.0)	
Housewife	266(27.0)	30(3.0)	296(30.1)	
Farmer	178(18.1)	18(1.8)	196(19.9)	
Business	134(13.6)	18(1.8)	152(15.4)	
Others	27(2.7)	0(0.0)	27(2.7)	

**Marital status**				0.87
Single	188(19.1)	30(3.0)	218(22.1)	
Married	621(63.0)	85(8.6)	706(71.7)	
Divorced/separated	18(1.8)	4(0.4)	22(2.2)	
Widowed	33(3.4)	5(0.5)	38(3.9)	
**Parity**				**<0.001***
Nullipara	219(22.2)	45(4.6)	264(26.8)	
Primipara	134(13.6)	10(1.0)	144(14.6)	
Two	115(11.7)	10(1.0)	125(12.7)	
Three	71(7.2)	18(1.8)	89(9.0)	
Four	97(9.8)	7(0.7)	104(10.6)	
Five	76(7.7)	15(1.5)	91(9.2)	
Six & Above (Multipara)	148(15.0)	17(1.7)	165(16.8)	

**Statistical significance at p-value <0.05*

Table 2 below assessed breast cancer awareness among our respondents; the majority (87.7%) had heard about breast cancer prior to the study and less than half (41.3%) reported mass media as their source of information. The majority of our participants identified obesity/overweight (35.2%), being a woman (35.0%), and having a family history of breast cancer (29.0%) as the common risk factors for breast cancer. However, less than 14.5% of our respondents knew about clinical breast examination and mammography (3.6%) as means of early detection and diagnosis of breast cancer. Based on breast cancer prevention, our respondents identified early initiation of breastfeeding, physical exercise, and limited alcohol intake (38.8%, 37.8%, and 23.8%, respectively) as measures to prevent breast cancer among females. Equally, nearly three-quarters (73.8%) were convinced that women of reproductive age should be screened for breast cancer.

Table 2 showing Breast cancer awareness of our respondents

variable	n	%
**heard about breast cancer (n=985)**		
Yes	864	87.7
No	121	12.3
**source of information**		
Family member	291	29.5
Health worker	305	31.0
Teacher	91	9.2
Friends	277	28.1
mass media	407	41.3
Others	48	4.9
**Knew Risk factors of breast cancer***		
Obesity/Overweight	125	35.2
Old age	164	16.6
Family history of breast cancer	293	29.0
Birth of first child after the age of 30 years	110	11.2

Early onset of menses (before age 12)	83	8.4
Late menopause (after 55 years)	49	5.0
Late initiation of breastfeeding	121	12.3
Being a woman	345	35.0
Cigarettee smoking/Alcohol consumption	221	22.4
Use of Oral contraceptive	82	8.3
Exposure to radiation	235	23.2
**knew breast examination methods***		
Breast self-examination	101	10.5
Clinical breast examination	147	14.5
Mammography	36	3.6
**Knew the preventive methods of breast cancer risk**		
Improve physical exercise	372	37.8
Initiation of breast feeding	382	38.8
Limit Alcohol intake	235	23.8
Avoid hormonal replacement therapy	115	11.7
**Knew who should be screen for breast cancer**		
Older women	404	41.0
Adolescents	619	62.8
Women in reproductive age	747	73.8
Pregnant women	485	49.2

**Multiple responses*

As shown in Figure 1, the majority of our respondents were aware that swelling of all parts of the breast(48.2%), nipple pain(45.2%), and lumps(37.1) were the major signs of breast cancer.

Only about 13% of our study participants reported having ever practiced any form of breast cancer screening, and 62.6% of those who had ever practiced breast cancer screening performed clinical breast examination as a method of breast cancer diagnosis. Only 1% of our respondents had a mammography done in their lifetime as a screening method. Breast pain was the sole reason for all those who had undergone breast screening, and nearly 60% of them also underwent screening based on the advice of a healthcare worker.

The major reason advanced by our respondents for not performing any breast cancer screening was the lack of knowledge (58.7%) on breast cancer screening methods and services. This is followed by service unavailability (13.5%) and the high costs (13.1%) associated with breast cancer screening.

Table 4 showing the practices of breast cancer screening

Variables		n	%
Have you ever practiced any breast cancer screening? (n=985)			
	Yes	124	12.6
	No	861	87.4
Which breast screening method? (n=124)*			
	Breast self-examination	49	39.5
	Clinical breast examination	78	62.6
	Mammography	14	1.1
Reasons for breast screening*			
	Breast pain	124	100
	Noticed a breast lump	50	40.3
	Advice from a health worker	73	58.9
Reasons for not breast screening (n=861)*			
	Lack of knowledge	505	58.7
	Forgetfulness	99	11.5
	Fear of the finding a mass	97	11.3
	Service unavailability	116	13.5
	High cost	113	13.1

**Multiple responses*

Predictors of breast cancer screening

Table 5 shows the predictors of breast cancer screening practices

The multivariate logistic regression analysis predicting breast cancer screening practices among women had several variables associated with screening practices, as shown in Table 5. Associations were found between ethnicity and breast cancer screening practices; serers were three times more likely to attend breast cancer screening compared to Mandinka, with an adjusted odds ratio (aOR= 2.821, 95% CI: 0.521 – 15.267).). In contrast, parity as whole was not associated with the uptake of breast cancer screening; however, categories of parity such as parity two, three, and five had decreased odds of breast cancer screening compared to nulliparity.

Associations were found between occupation and breast cancer screening practises; civil servants had an increased odds of breast cancer screening by a factor of more than two compared to unemployed women (aOR= 2.778, 95% CI: 1.174 – 6.573). Consistently, students also had three times the likelihood of breast cancer screening practice compared to unemployed women (aOR= 3.111, 95% CI: 1.453 – 6.663).

**Variables**	**aOR**	**95%CI**	**p-value**
**Ethnicity**			0.005*
Mandinka	1		
Fula	1.552	0.872 - 2.761	0.135
Wolof	1.559	0.977 - 2.486	0.062
Serahuli	1.359	0.541 - 3.387	0.517
Jola	0.867	0.188 - 3.990	0.854
Serer	2.821	0.521 - 15.267	0.229

**Parity**			0.529
Nullipara	1		
Primipara	0.12	0.10 - 1.448	0.095
Two	0.061	0.005 - 0.791	0.032*
Three	0.075	0.006 - 0.967	0.047*
Four	0.238	0.019 - 3.021	0.269
Five	0.065	0.005 - 0.877	0.040*
Six & Above (Multipara)	0.196	0.015 - 2.513	0.211
**Current Occupation**			0.005*
Unemployed	1		
Civil servant	2.778	1.174 - 6.573	0.020*
Student	3.111	1.453 - 6.663	0.003*
Housewife	1.022	0.489 - 2.135	0.954
Farmer	0.936	0.413 - 2.122	0.875
Business	1.138	0.506 - 2.560	0.755

**Statistical significance $p<0.05$, aOR, Adjusted Odds Ratio*

Discussion

To attain the target set by the WHO through the Global Breast Cancer Initiative (GBCI) in The Gambia, there is a need to target all three 3 priority areas for the reduction of both morbidity and mortality from breast cancer. Our study examined breast cancer level of awareness and current screening practices in rural Gambian women. The study focused mainly on assessing the knowledge of breast cancer signs and symptoms, risk factors, prevention and control of breast cancer, and medical screening practices to diagnose breast cancer through breast self-examination, clinical breast examination, and mammography.

Our findings revealed that nearly 9 out of ten rural women knew the term breast cancer before our study. This finding is similar to a study conducted by Kinteh et al. among female University Students, where all respondents heard of breast cancer before the study and a study conducted in Gaza, where more than 80% of respondents also heard about breast cancer (Abo Al-Shiekh et al., 2021). Our findings on breast cancer knowledge were 3 times higher than that of a study done in a rural South African setting where only 31% of respondents were aware of breast cancer (Ramathuba et al., 2015) and a study done in Ethiopia where almost half (49.87%) had never heard of self-breast-examinations (Assfa Mossa, 2022).

Mass media serve as an efficient and effective means of disseminating information to people, and they are instrumental in health promotion and education. More than 40% of our respondents had heard about breast cancer through media programs. This is similar to research done in Ethiopia where 57.91% heard of breast cancer through Television/Radio (Assfa Mossa, 2022) and in Addis Abba, 70.10% heard about breast cancer from TV/Radio(Tewelde et al., 2022). The Directorate of Health Promotion and Education under the Ministry of Health of The Gambia is a media-friendly centre that is responsive to current health issues and is proactive in educating the population on common health problems in the country.

Our respondents demonstrated an average knowledge of breast cancer risk factors. They correctly identified obesity/overweight (35.2%), female sex (35.0%), family history (29.0%), and cigarette smoking/alcohol consumption (22.4%) as risk factors for breast cancer. This

finding is similar to a study done by (Ramathuba et al., 2015)in South Africa which showed the use of oral contraceptives and late menopause as less common risk factors. Similarities were also found in work done in Nigeria where 11.8% and 18.7% of respondents reported late menopause and birth control pill use as risk factors for breast cancer (Ifediora & Azuike, 2018). The country has low contraceptive uptake among reproductive women (19%)(GBoS, 2020) and as such, it translates to limited knowledge of oral contraceptives as a risk factor for breast cancer among our respondents. Conversely, knowledge about late menopause as a risk factor is lower than that reported in many studies in Sub-Saharan Africa(Assfa Mossa, 2022; Ramya Ahmad et al., 2019).

Our study identified a huge knowledge gap regarding breast cancer screening methods and diagnostics. Less than 15% of our respondents knew about clinical breast examinations, 10% knew about breast self-examinations, and up to 4% knew about mammography. This finding corroborates the spatial availability of breast cancer diagnostic services in the country as reported by Sanyang and his colleagues(Sanyang et al., 2021). Efforts should be centred on improving breast cancer education including knowledge of self-breast examinations and diagnostic methods that could improve the screening practices of women.

Breast cancer prevention among women is a global target desirable for all stakeholders. Our respondents advanced the initiation of breastfeeding (38.8%) and improved physical exercise (37.8%) as methods of preventing breast cancer in women. These are among the WHO-recommended preventive methods for breast cancer among women(WHO, 2024).

Our respondents' awareness of the tell-tale signs of symptoms of breast cancer aligned around four major signs: swelling of the breast (48.2%), nipple pain(45.2%), breast lump (37.1%)p, and sores on the breast (26.8%). These findings are similar to studies conducted among women in Qatar, Southwest Ethiopia, and Northwest Ethiopia(Asmare et al., 2022; Assfa Mossa, 2022; Hamed et al., 2022). However, there is limited awareness among our respondents regarding the signs of nipple discharge, retracted nipple, change in breast colour, scaling, and thickening of the nipple, and efforts are needed to raise more awareness.

The practice of screening for breast cancer was low among our respondents, as less than 13% of them had ever had any form of clinical breast examination. Among those who have screened for breast cancer, more than 62% of them practised clinical breast examination. Mammography is the least practised (1.1%) breast cancer screening method among our respondents because of the unavailability of this screening method in rural areas, as evidenced by Sanyang et al., who found that breast cancer Histopathological diagnosis and surgical management remain unavailable to over 50% of the population in Gambia(Sanyang et al., 2021). Breast Self-Examination, which is a simple and cost-free method of screening for breast diagnosis, is practised by less than 40% of our respondents. This finding is lower than those reported in studies conducted in Uganda (76.5%), Cameroon (47.0%), Northwest Ethiopia (45.8%), and Addis Ababa (43.6%) (Asmare et al., 2022; Godfrey et al., 2016; Sama et al., 2017; Tewelde et al., 2022). However, the practice of breast self-examination is higher than that of a similar study conducted in Southern parts of Ethiopia (21.1%)(Israel et al., 2023). Variability in breast self-examination among countries could be associated with social and literacy differences. Eventually, breast pain was the reason for the practice of breast cancer screening, and lack of knowledge was a major barrier to breast cancer screening in this study.

In this study, women with a parity of two were 6% less likely to undergo breast cancer screening than those with nulliparity [AOR: 0.061, 95% CI: 0.005 – 0.791)]. Additionally, women with a parity of three were 7% less likely to undergo breast cancer screening [AOR, 0.075; 95% CI, 0.006 – 0.967)]. Furthermore, women who had a parity of five were also 6% less likely to undergo breast cancer screening compared to nulliparity [AOR: 0.065, 95% CI: 0.005 – 0.877)]. An increase in parity showed a decrease in the practice of breast cancer screening among our respondents. Massive education and sensitization are needed for rural women to undergo breast cancer screening.

The current occupation of our respondents is a predictor of breast cancer screening practice; civil servants have a factor of nearly three times to practice any breast cancer screening practices as compared to unemployed women [AOR: 2.778, 95% CI: 1.174 – 6.573)]. The practice of breast cancer screening among civil servants is directly influenced by their socioeconomic status. This finding is indirectly variable to studies where educated participants are more likely to get screened for breast cancer (Asmare et al., 2022; Israel et al., 2023). In addition, students were more likely to get screened for breast cancer screening than unemployed women [AOR: 3.111, 95% CI: 1.453 – 6.663)].

Strengths and Limitations of the study

The findings of this study could be generalized to the rural female population of the country as there is no distinct sociodemographic difference between North and South Gambia regions. Additionally, the study was conducted among women in rural areas with some hard to reach communities involved in the study depicting the real status of breast cancer screening among them.

As a cross-sectional study, the study could not ascertain the causal between dependent and independent variables and potential information bias could have occurred during interviews.

Conclusion

This study revealed that rural women's awareness of breast cancer was above average, as they could correctly mentioned the signs and symptoms of breast cancer, risk factors, and mode of prevention of the disease. Breast cancer screening practice was, however, found to be below that of many sub-Saharan African studies. The predictors of breast cancer screening practices were influenced by the parity of the women and the current occupation of the respondents. However, we recommend that MOH and its partners improve awareness creation among rural women with breast cancer. Additionally, breast cancer screening services, such as clinical breast examination and screening mammography, should be made available, accessible and affordable to rural women to ensure early detection and timely interventions to save lives.

Acknowledgement

We acknowledge the contributions the Students of Public Health, at Gambia College and Medical Students from the University of The Gambia made during the data collection. Their dedication and services during this period contributed to the success of the research. We also wish to acknowledge our respondents for their active participation during the data collection.

Conflict of interest

The authors declare and affirm that they do not have any personal, financial, or professional interests that could potentially influence or tamper with the validity of the results. This work is therefore free of biases that could compromise its scientific integrity.

Author contributions

The authors of this work have been actively involved and have made substantial contributions to every aspect from conceptualization and literature review to the data analysis to manuscript writing and review of final work for publication. Every author has carefully reviewed and approved the final manuscript for publication.

Consent

Participation in the study was voluntary and only individuals who provided consent were recruited. All participants who agreed to participate in the study provided verbal informed consent.

References

Abo Al-Shiekh, S. S., Ibrahim, M. A., & Alajerami, Y. S. (2021). Breast Cancer Knowledge and Practice of Breast Self-Examination among Female University Students, Gaza. The Scientific World Journal, 2021, 1–7. https://doi.org/10.1155/2021/6640324

Arnold, M., Morgan, E., Rumgay, H., Mafra, A., Singh, D., Laversanne, M., Vignat, J., Gralow, J. R., Cardoso, F., Siesling, S., & Soerjomataram, I. (2022). Current and future burden of breast cancer: Global statistics for 2020 and 2040. The Breast, 66, 15–23. https://doi.org/10.1016/j.breast.2022.08.010

Asmare, K., Birhanu, Y., & Wako, Z. (2022). Knowledge, attitude, practice towards breast self-examination and associated factors among women in Gondar town, Northwest Ethiopia, 2021: A community-based study. BMC Women's Health, 22(1), 174. https://doi.org/10.1186/s12905-022-01764-4

Assfa Mossa, K. (2022). Perceptions and knowledge of breast cancer and breast self-examination among young adult women in southwest Ethiopia: Application of the health belief model. PLOS ONE, 17(9), e0274935. https://doi.org/10.1371/journal.pone.0274935

Espina, C., McKenzie, F., & dos-Santos-Silva, I. (2017). Delayed presentation and diagnosis of breast cancer in African women: A systematic review. Annals of Epidemiology, 27(10), 659-671.e7. https://doi.org/10.1016/j.annepidem.2017.09.007

GBoS. (2013). Distribution of Population by Gender and LGa. Gambia Bureau of Statistics. https://www.gbosdata.org/topics/population-and-demography/percentage-distribution-of-the-population-by-lga-a/download/pdf

GBoS. (2020). The Gambia Bureau of Statistics (GBoS), Ministry of Health (MoH) [The Gambia], and ICF. 2020. The Gambia Demographic and Health Survey 2019-20: Key Indicators Report. Banjul, The Gambia and Rockville, Maryland, USA: The Gambia Bureau of Statistics, Ministry of Health, and ICF.

Globocan. (2021). The Republic if The Gambia Fact sheets. https://gco.iarc.fr/today/data/factsheets/populations/270-the-republic-of-the-gambia-fact-sheets.pdf

Godfrey, K., Agatha, T., & Nankumbi, J. (2016). Breast Cancer Knowledge and Breast Self-Examination Practices Among Female University Students in Kampala, Uganda: A Descriptive Study. Oman Medical Journal, 31(2), 129–134. https://doi.org/10.5001/omj.2016.25

Hamed, E., Alemrayat, B., Syed, M. A., Daher-Nashif, S., Rasheed, H. M. A., & Kane, T. (2022). Breast Cancer Knowledge, Attitudes and Practices amongst Women in Qatar. International Journal of Environmental Research and Public Health, 19(7), 3995. https://doi.org/10.3390/ijerph19073995

Ifediora, C. O., & Azuike, E. (2018). Tackling breast cancer in developing countries: Insights from the Knowledge, Attitudes and Practices on breast cancer and its prevention among Nigerian teenagers in secondary schools. Journal of Preventive Medicine and Hygiene, Vol 59, E282 Pages. https://doi.org/10.15167/2421-4248/JPMH2018.59.4.964

Israel, E., Awoke, N., Yakob, T., Aynalem, A., Talto, A., & Bezabih, K. (2023). Determinants of breast self-examination practice among women attending pastoralist health facilities, Southern Ethiopia: A cross-sectional study. BMC Women's Health, 23(1), 14. https://doi.org/10.1186/s12905-023-02158-w

Kassie, A. M., Abate, B. B., Kassaw, M. W., & Shiferaw, W. S. (2021). Breast Self-Examination Practice Among Female University Students in Ethiopia: A Systematic Review and Meta-Analysis. Cancer Control, 28, 107327482110191. https://doi.org/10.1177/10732748211019137

Kinteh, B., Kinteh, S. L. S., Jammeh, A., Touray, E., & Barrow, A. (2023). Breast Cancer Screening: Knowledge, Attitudes, and Practices among Female University Students in The Gambia. BioMed Research International, 2023, 1–8. https://doi.org/10.1155/2023/9239431

Kohler, R. E., Gopal, S., Miller, A. R., Lee, C. N., Reeve, B. B., Weiner, B. J., & Wheeler, S. B. (2017). A framework for improving early detection of breast cancer in sub-Saharan

Africa: A qualitative study of help-seeking behaviors among Malawian women. Patient Education and Counseling, 100(1), 167–173. https://doi.org/10.1016/j.pec.2016.08.012

Ministry of Health. (2021). Health Policy 2021—2030.

Pace, L. E., & Shulman, L. N. (2016). Breast Cancer in Sub-Saharan Africa: Challenges and Opportunities to Reduce Mortality. The Oncologist, 21(6), 739–744. https://doi.org/10.1634/theoncologist.2015-0429

Pal, A., Taneja, N., Malhotra, N., Shankar, R., Chawla, B., Awasthi, A., & Janardhanan, R. (2021). Knowledge, attitude, and practice towards breast cancer and its screening among women in India: A systematic review. Journal of Cancer Research and Therapeutics, 17(6), 1314. https://doi.org/10.4103/jcrt.JCRT_922_20

Ramathuba, D. U., Ratshirumbi, C. T., & Mashamba, T. M. (2015). Knowledge, attitudes and practices toward breast cancer screening in a rural South African community. Curationis, 38(1), 8 pages. https://doi.org/10.4102/curationis.v38i1.1172

Ramya Ahmad, S., Asmaa Ahmad, A., Nesreen Abdullah, A., Rana Ahmad Bin, S., Shaimaa Amer, A., Aisha, T., & Mohammad Shahid, I. (2019). Awareness Level, Knowledge and Attitude towards Breast Cancer between Medical and Non-Medical University Students in Makkah Region: A Cross Sectional Study. International Journal of Cancer and Clinical Research, 6(1). https://doi.org/10.23937/2378-3419/1410106

Sama, C.-B., Dzekem, B., Kehbila, J., Ekabe, C. J., Vofo, B., Abua, N. L., Dingana, T. N., & Iii, F. A. (2017). Awareness of breast cancer and breast self-examination among female undergraduate students in a higher teachers training college in Cameroon. Pan African Medical Journal, 28. https://doi.org/10.11604/pamj.2017.28.91.10986

Sanyang, O., Lopez-Verdugo, F., Mali, M., Moustafa, M., Nellermoe, J., Sorensen, J., Bittaye, M., Njie, R., Singhateh, Y., Sambou, N. A., Goldsmith, A., Mohammed, N. I., Brownson, K. E., Price, R. R., & Sutherland, E. (2021). Geospatial analysis and impact of targeted development of breast cancer care in The Gambia: A cross-sectional study. BMC Health Services Research, 21(1), 943. https://doi.org/10.1186/s12913-021-06963-7

Tewelde, B., Tamire, M., & Kaba, M. (2022). Breast self-examination practice and predictors among female secondary school teachers in Addis Ababa, Ethiopia: Using the health belief model. BMC Women's Health, 22(1), 317. https://doi.org/10.1186/s12905-022-01904-w

WHO. (2024). Breast cancer. https://www.who.int/news-room/fact-sheets/detail/breast-cancer

Figure

Click here to access/download;Figure;Figure 1.tif

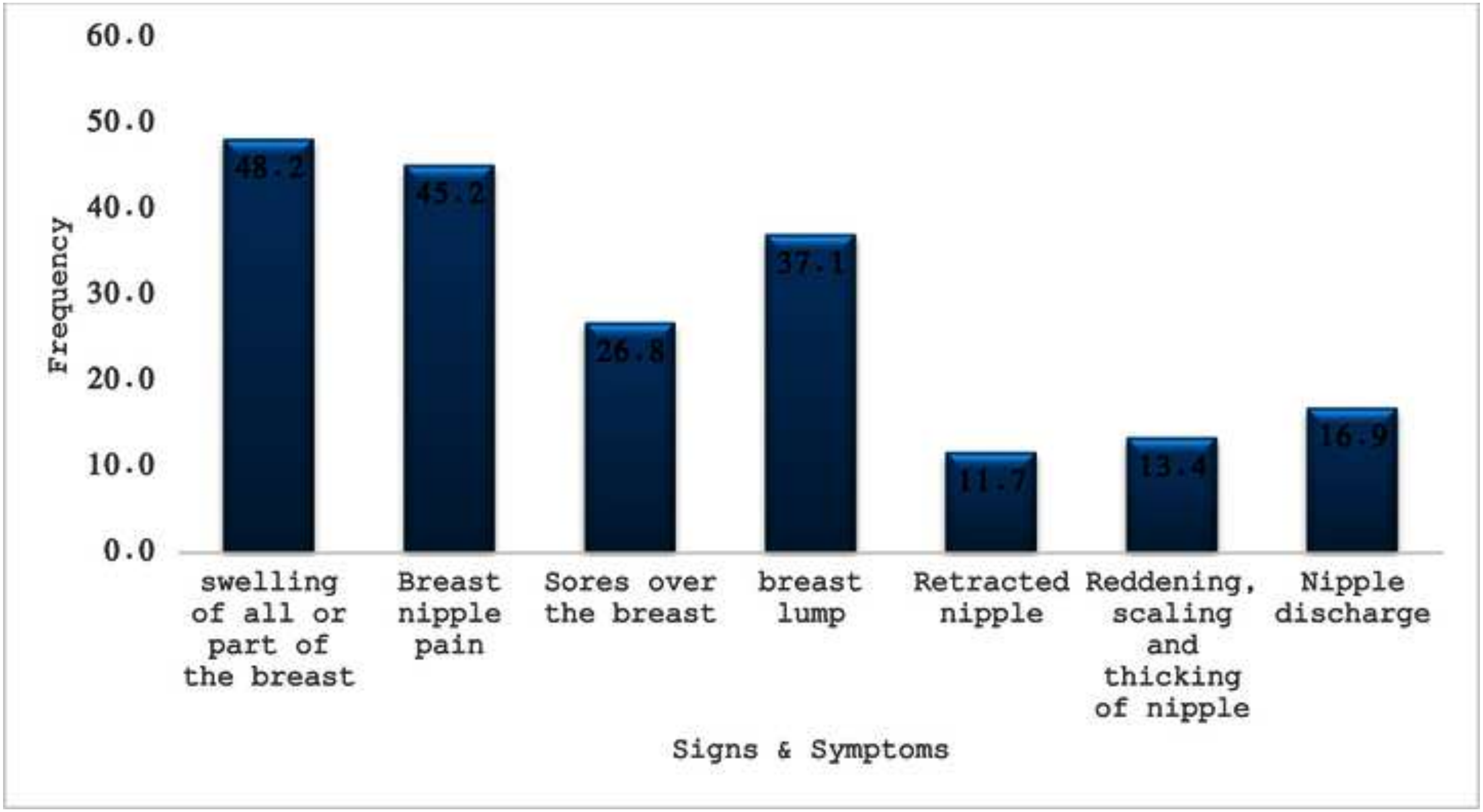

Supplement: Attachment — Submitted filename: PONE-D-24-19264.pdf [file pone.0307785.s003.pdf]
