# Supplementary material for: Exploring breast cancer awareness and screening practices amongst rural women in The Gambia: Community-based cross-sectional study
Source: PLoS One. 2025 May 12;20(5):e0307785. doi: 10.1371/journal.pone.0307785 (PMC12068578; doi:10.1371/journal.pone.0307785)
Supplement: Attachment — Submitted filename: PONE-D-24-19264_RFA.pdf [file pone.0307785.s005.pdf]

General comment

The manuscript has improved considerably. However, I still a bit confused on the main outcome of interest operational definition. To my understanding, the terms "breast cancer screening practices" and "breast cancer screening uptake" refer to different aspects of breast cancer screening. While screening practices are about the methods and guidelines for conducting screenings, uptake relates to how many people actually get screened. Nonetheless, exploring the two concepts is essential at improving breast cancer prevention efforts and achieving better health outcomes. I therefore advise that the two concepts be clearly delineated in the body of the manuscript, as clearly stated in the objective of the study (Lines 24-26).

Specific comments

Title:

- If word the "Northern" will not appear on the title, I suggest how the region was selected be clearly mentioned under the sampling strategy subsection of the Method section.

Abstract:

- Methods:
  - Line 28: I suggest the word Northern be deleted if it will not be retained in the title as commented above
  - Line 31: As explained under the general statement, I suggest the phrase "breast cancer screening practices" be replaced with "**breast cancer screening uptake**"
  - Lines 32-33: authors should recast …
  - Provide a statement to indicate that adjusted odds ratios was reported in the Results section
- Results:
  - aOR – adjusted Odds Ratio is mentioned without being defined at first use. This could be avoided if the comment above is addressed.

- Conclusions:
  - Line 45: what makes it suboptimal ... the use of the statement is unclear
  - Lines 45-46: The statement "The key barriers include knowledge deficits, service unavailability, and cost constraints" could not be linked with any of the result presented in the Results section of the abstract.

Introduction:

  - Lines 85-86: Authors should recast the objective statement to include the screening uptake

Methods:

- **Study design and setting**
  - Lines 94: the phrase … northern region of … should be addressed as commented earlier
  - Line 105: I suggest the deletion of the phrase "Healthcare Access and Barriers"
  - Lines 105-115: There is a need to reference some of the factual statements in the paragraph

- **Study population and eligibility criteria**
  - Lines 120-125: Authors are advised to delete the exclusion statement in lines 123-124, as exclusion criteria statement is NOT expected to be opposite of inclusion criteria statement. Expctedly, exclusion criteria statement should addressed only women that would not be eligible out of the women already included in the study
- **Study variables**
  - Line 127: As mentioned earlier, the main outcome variable was the "breast cancer screening uptake". This should be reflected in other sections of the study. The evidence is clear from the analysis.

- **Sampling strategy**
  - Lines 152-166: The first two stages, as mentioned here, are not selection stages. The selected women were from the two LGAs with a total of seven districts, in the northern region of The Gambia.
  - As I explained earlier, if Northern will not be included in the title, I advise the selection of the Northern region be the first selection stage. Is it selected purposively or by any other method?
  - If the word northern is included in the title, the 3rd stage should become the 1st stage, in that order.

- **Data management and statistical analysis**
  - Lines 194-196: replace the statement with "Descriptive statistics such as means with standard deviations (SD) for continuous variables, and frequencies and percentages for categorical variables were used present the women characteristics including their breast cancer awareness and screening practices".
  - Line 198: replace "practices" with "uptake"
  - Line 225: The clause "and statistical significance was declared at $p<0.05$." may be deleted as the statement is already implied by the use of 95% confidence interval.

Results:

- Line 237: Sociodemographic characteristics … of what??? I suggest "Women's sociodemographic characteristics" as the subsection title
- Lines 238-239: The statement "Of the 1,012 women recruited from thirty-five communities, 985 completed the study (response rate: 97.3%)." is not a result. Authors should delete the statement. Besides, it is clearly mentioned under the Methods section

- Figure 1: delete the figure, but ensure a total row (last row) is created in Table 1
- Line 251: replace the subsection with "Association between the selected women's characteristics and breast cancer screening uptake",
  - Importantly, this should be presented after the beast cancer awareness and practices descriptive presentations.
- Table 1
  - I quite understand that arrangement of table's columns, including row-wise/column-wise percentages presentation, does not influence the chi-square result. However, for sake of clarity to the potential Plos One reader, my previous

suggestion may be necessary, especially in relation to the statement in lines 256-258.
    - Additionally, I suggest that the title should read as “Distribution of women’s socio-demographic characteristics by breast cancer screening uptake
- Lines 265-275: The paragraph should have a subsection title “**Breast cancer awareness**”
- Lines 279-280: delete the statement on breast cancer screening uptake. As suggested earlier, Table 1 should be sufficient to indicate it.
- Figure 2: should be renamed Figure 1
    - It will be nice if the figure is reproduced in descending order of percentage magnitudes
    - Its title may be replaced with “Distribution of breast cancer’s signs and symptoms”
    - It may be placed as an appendix
- Table 4 – correct as Table 3
    - Its title may be replaced with “Distribution of breast cancer screening practices”
    - Delete first items “Have you ever practiced any breast cancer screening?”, to avoid repletion of information
- Line 301: replace with “**Predictors of breast cancer screening uptake**”
- Table 4
    - Replace the title with “Adjusted Odds Ratios of breast cancer screening uptake by women’s selected characteristics”
- **Discussion**
    - Line 335: breast cancer knowledge??? A composite score would be needed to speak to this, which is not included in the work. Of course, the focus is not on knowledge; authors are enjoined to avoid the mix-up between awareness and knowledge.
