# Supplementary material for: Exploring breast cancer awareness and screening practices amongst rural women in The Gambia: Community-based cross-sectional study
Source: PLoS One. 2025 May 12;20(5):e0307785. doi: 10.1371/journal.pone.0307785 (PMC12068578; doi:10.1371/journal.pone.0307785)
Supplement: Attachment — Submitted filename: PONE-D-24-19264R2_RFA.pdf [file pone.0307785.s007.pdf]

The responses to the comments are appreciated. However, I would like to see the following comments addressed in order to further enriched the manuscript.

Specific comments

Abstract:

- Background:
  - Lines 21-23: replaced with the statement “This study aimed to assess breast cancer awareness and identify associated factors influencing screening uptake among rural women in The Gambia”
- Methods:
  - Line 25: replace the word “Gambia” with “The Gambia”, and elsewhere in the manuscript
  - Line 29: replace the phrase “Chi-squared and/or Fisher’s exact test” with “Chi-squared or Fisher’s exact test, as appropriate”
  - Line 29: replace the phrase “were reported was in the results.” with “were reported”
- Results:
  - Line 36: replace the phrase “Multivariable analysis revealed that students” with “Students”
- Conclusions:
  - Lines 43-44: Authors are advised to include result(s) suggesting the phrase “primarily due to limited knowledge of the urgent need for accessible and affordable screening services” in the Results section of the abstract
  - Lines 44-45: Let the recommendation statement be presented in a separate sentence.

Material and Methods:

- **Study design and setting**
  - Lines 94: the phrase … northern region of … should be addressed as commented earlier
  - Line 105: I suggest the deletion of the phrase “Healthcare Access and Barriers”
  - Lines 105-115: There is a need to reference some of the factual statements in the paragraph

- **Study population and eligibility criteria**
  - Line 119: replace with “**Study population**”
- **Sample size determination**
  - Lines 146-148: Authors should ensure the correctness of the following stated figures!
    1. Estimated minimum sample size becomes **1103** (not 1092 as indicated) if adjusted for 10% non-response with 1.5 design effect
    2. Besides, let’s assume 1092 is correct! Then, only of **1012** (not 1092) that 97.3% response rate would yield 985 completed the study

- **Sampling strategy**

- Lines 151-162: replace with "A four-stage sampling method was employed. First, the northern region was purposively selected due to its geographical location, high population density of underserved rural women, and absence of mammography services. This selection aligns with the study's aim of understanding screening uptake in rural, as it represents a significant proportion of rural communities with limited healthcare access. The northern region of The Gambia comprises of Kerewan and Kuntaur local government areas (LGAs), with four (Upper Baddibu, Lower Baddibu, Central Baddibu, and Jokadu) and three (Upper Saloum, Lower Saloum, and Niani) districts, respectively. Second, five communities were randomly selected from each district, yielding 35 study communities in total. Third, within each selected community, households were sampled using probability proportional to size, selecting an average of **25-35** households per community. Finally, one eligible woman was selected from each household."
- Also important to note, selection of 25-30 households in each of the 35 communities would not yield the minimum sample size estimated (30*35=1050; this cannot lead to 1092 assuming its correct). However, **25-35** households per community will do!

- **Data management and statistical analysis**
  - Lines 197-199: The women age was not covered with the present statement. I refer the authors to my previous advice to replace the statement with "Descriptive statistics such as means with standard deviations (SD) for continuous variables, and frequencies and percentages for categorical variables were used present the women characteristics including their breast cancer awareness and screening practices".
  - Line 199: replace "We employed Pearson's chi-squared and/or Fisher's exact test to" with "As appropriate, Pearson's chi-squared or Fisher's exact test was used to … "
  - Lines 204-207: Authors are advised to place the statement "Despite its non-significance at the bivariate analysis level, ethnicity was retained in the model owing to its theoretical importance and our specific interest in understanding its effects on breast cancer screening uptake in relation to other predictors" immediate on line 211 after "bivariate level." to facilitate a smooth narrative.

Results:

- Figure 1:
  - Line 251: replace "practices" with "uptake"
- Table 1
  - For sake of clarity to the potential Plos One readers, my previous suggestion is necessary!
    - Column 2 to be presented as Total (column percentage), while others remain as they were
    - By so doing, statements on women's characteristic in lines 240-247 could be referenced adequately.
  - Additionally, I suggest that the title should read as "Distribution of women's socio-demographic characteristics by breast cancer screening uptake

- Lines 291: I suggest the paragraph subsection title be read as "**Breast cancer screening practices**" as this includes BC signs and symptoms.
- Lines 299-302: The statement "These findings highlight the complex interplay of structural, knowledge-based, and personal barriers influencing breast cancer screening uptake in this rural population, suggesting the need for targeted interventions to address these multiple barriers to screening." should be moved under Discussion section

Thank you!

- The terms "breast cancer screening practices" and "breast cancer screening uptake" refer to different aspects of breast cancer screening.
-
- 1. **Breast Cancer Screening Practices**: This term generally refers to the recommended approaches and methods used to screen for breast cancer. It includes guidelines about how often screenings should be performed (like mammograms), the age at which women should begin screening, the types of screening available (such as clinical exams and self-exams), and best practices for implementing these screenings in healthcare settings.
-
- 2. **Breast Cancer Screening Uptake**: This term specifically refers to the extent to which eligible individuals participate in breast cancer screening programs. It’s a measure of how many women are actually getting screened compared to those who are advised to do so according to the established practices. Uptake can be influenced by various factors such as awareness, accessibility, socioeconomic status, and cultural beliefs.
-
- In summary, breast cancer screening practices focus on the guidelines and methods for screening, while breast cancer screening uptake measures the participation rates in those practices.

"Breast cancer screening practices" and "breast cancer screening uptake" refer to different but related concepts in the context of breast cancer prevention and detection.

1. **Breast Cancer Screening Practices**: This term encompasses the various methods, guidelines, and recommendations regarding how breast cancer screening is conducted. It includes the screening techniques (like mammography, clinical breast exams, and self-exams), the frequency and age recommendations for screenings, the protocols for providing information to patients, and the healthcare provider's role in facilitating screenings. Practices are influenced by clinical guidelines, public health recommendations, and factors like availability of resources and technology.

2. **Breast Cancer Screening Uptake**: In contrast, screening uptake refers to the extent to which eligible individuals participate in these screening practices. It is often measured by the proportion of the target population that undergoes screenings within a specified time frame. Factors affecting uptake include awareness, accessibility, socioeconomic status, cultural beliefs, and healthcare systems. High uptake indicates that a larger percentage of the target population is participating in recommended screenings, while low uptake may indicate barriers or lack of awareness.

In summary, while screening practices are about the methods and guidelines for conducting screenings, uptake relates to how many people actually get screened. Monitoring both aspects

is essential for improving breast cancer prevention efforts and achieving better health outcomes. If you have further questions or need more specific examples, feel free to ask!
