# Supplementary material for: Exploring breast cancer awareness and screening practices amongst rural women in The Gambia: Community-based cross-sectional study
Source: PLoS One. 2025 May 12;20(5):e0307785. doi: 10.1371/journal.pone.0307785 (PMC12068578; doi:10.1371/journal.pone.0307785)
Supplement: Attachment — Submitted filename: PONE-D-24-19264R3_RFA.pdf [file pone.0307785.s009.pdf]

The authors are commended for their efforts in improving this manuscript. The revisions are largely satisfactory; however, a few comments appear to have been addressed inappropriately, likely due to a misunderstanding of their intent. To ensure clarity, I have relisted those comments below for your reference

**Abstract:**

- Conclusions:
  - *Lines 43-44: Authors are advised to include result(s) suggesting the phrase "primarily due to limited knowledge of the urgent need for accessible and affordable screening services" in the Results section of the abstract*

- The comment requests that the authors report the reasons for the poor uptake of breast cancer screening. Simply referencing "limited knowledge" without presenting supporting data in the Results section would be inappropriate.
- Besides, it is not necessary to include those percentages under the Conclusion section of the abstract. It is better reported under the Results section.

**Sample size determination**

- *Lines 146-148: Authors should ensure the correctness of the following stated figures!*
  1. *Estimated minimum sample size becomes **1103** (not 1092 as indicated) if adjusted for 10% non-response with 1.5 design effect*
  2. *Besides, let's assume 1092 is correct! Then, only of **1012** (not 1092) that 97.3% response rate would yield 985 completed the study*

- Regardless of the statistical software used for sample size computation, the minimum sample size of 662 is accurate based on the presented indices. However, when accounting for the design effect, the minimum sample size increases to 993 (662 * 1.5). If we then adjust for a 10% non-response rate, the required sample size becomes 1103 (993 * 0.9).
- Authors are requested to address this appropriately!

**Results:**

- Table 1
  - *For sake of clarity to the potential Plos One readers, my previous suggestion is necessary!*
    - *Column 2 to be presented as Total (column percentage), while others remain as they were*
    - *By so doing, statements on women's characteristic in lines 240-247 could be referenced adequately.*
- The comments request that the authors include column percentages for the Total column. To avoid any further confusion, I suggest renaming the Total column heading to "n (%)" so that the reported percentages are clearly indicated on a column-wise basis (NOT 100, as presently reported).

Thank you!
