# Supplementary material for: Exploring breast cancer awareness and screening practices amongst rural women in The Gambia: Community-based cross-sectional study
Source: PLoS One. 2025 May 12;20(5):e0307785. doi: 10.1371/journal.pone.0307785 (PMC12068578; doi:10.1371/journal.pone.0307785)
Supplement: Attachment — Submitted filename: PLOS ONE REVIEWERS COMMENTS.docx [file pone.0307785.s001.docx]

**PLOS ONE	REVIEWER’S COMMENTS**

**Topic: Exploring Breast Cancer Awareness and Screening Practices amongst Gambian Women in the Gambia; Community-based- cross-sectional study**

**Dear Editor PLos One Journal,**

Many thanks for the opportunity to review this paper from the Gambia. I am grateful.

**General Comments**

A good and interesting paper. It adds to the breast cancer literature from the Gambia. However, the topic needs revision.

The background outlined the key issues related to breast cancer awareness and screening practices in the Gambia

The statistical analysis were appropriate

The findings presented were relevant and the discussion gave an in-dept explanation on the topic.

Conclusion: the conclusion is generally sound and based on the findings, however, it needs a slight revision.

**Specific Comments**

1. **Tropic:** One of the study variables looked at nationality; Gambian and non Gambian. Therefore the authors should consider removing the term Gambian on the topic. The proposed topic should read“ **Exploring Breast Cancer Awareness and Screening Practices amongst Women in the Gambia; Community-based- cross-sectional study** ”
2. What was the justification to choose simple random sampling to select participants in households with more than a single eligible respondent on line 130-131? Secondly, how were/was household(s) with no eligible respondent dealt with in the study?
3. Were the research assistant from Solace foundation trained for the data collection at the community level?
4. Table 1 and table 2, authors were using participants and in other tables respondents. They should be consistent with the terms. Table 2 should read “Breast cancer awareness of respondents”
5. Lines 202 and 207 the term “our respondents” should be change to “the respondents” all throughout the document.
6. Table 2 all variables with multiple respondents should be indicated eg “ Knew the preventive methods of breast cancer risk”
7. Line 239 Kinteh et al , no year was added to the reference. Similar to Sanyang et al line 290. Therefore it should be rephrased
8. Conclusion Line 330-332- “*The predictors of breast cancer screening practices were influenced by the parity of the women and the current occupation of the respondents” .* Do they mean ethnicity? Parity was not, even though some of its sub categories indicated so.

Yours Sincerely

Jainaba Sey-Sawo (Ph.D.)
