# Supplementary material for: Exploring breast cancer awareness and screening practices amongst rural women in The Gambia: Community-based cross-sectional study
Source: PLoS One. 2025 May 12;20(5):e0307785. doi: 10.1371/journal.pone.0307785 (PMC12068578; doi:10.1371/journal.pone.0307785)
Supplement: Attachment — Submitted filename: Breast cancer review.docx [file pone.0307785.s002.docx]

The article provides interesting findings on breast cancer in The Gambia. I think the paper is worthy of publication, but I have a few questions and suggestions for improvement with regard to the methodology and the evaluation strategy.

The authors should better justify the statistical model they chose with mathematical expressions for the models. The descriptive results are not sufficient, charts can be used to support the results.

Although, the article demonstrated scholarly argument, the article is poorly written with a lot of typos and grammatical errors. The paper needs some editing and proof-reading as well as some double-check for consistency in spelling ( I have indicated some out of so many).

**Accept with major revision**
