# Supplementary material for: Exploring breast cancer awareness and screening practices amongst rural women in The Gambia: Community-based cross-sectional study
Source: PLoS One. 2025 May 12;20(5):e0307785. doi: 10.1371/journal.pone.0307785 (PMC12068578; doi:10.1371/journal.pone.0307785)
Supplement: Attachment — Submitted filename: Response to reviewers.docx [file pone.0307785.s006.docx]

November 14, 2024

Manuscript ID: [PONE-D-24-19264] - [EMID:b560eba95e797023]

**Title:** “Exploring Breast Cancer Awareness and Screening Practices Amongst Rural Women in The Gambia: Community-based Cross-sectional Study”

RE: Revised manuscript submission and response to reviewers’ comments

Dear Editor,

This letter is in reference to your email dated November 5, 2024 with reviewers’ comments. We are very pleased that the manuscript is potentially acceptable for publication in *PLOS ONE* once we have carried out the revisions.

We would like to thank the reviewers for these insightful and helpful comments and for giving us the chance to revise our manuscript. We believe the revised manuscript has been significantly improved and the reviewers’ comments have been addressed adequately. We think in its current form it will make a valuable contribution to the literature on this increasingly important topic. 

Please find for your kind consideration the following:

- A section-by-section response to the comments and suggestions of the reviewers (below).
- The revised manuscript, provided as a marked-up copy and a clean copy.

We hope that these changes meet with your favourable consideration. Please do not hesitate to get in touch if you require any further information. 

Bakary Kinteh
Corresponding Author 

Reviewer #1:

The authors have described findings from of breast cancer awareness and screening practices among rural Gambian women in a community-based cross-sectional study. Although awareness was high with 87.7% of women being aware of breast cancer, only 13% practised screening.
Major issues;
1-The distance or proximity of the closest medical facility to the subjects. What kind/level of service are they able to receive, when available?

**Response**: The specifics of available services are beyond the scope of this paper. We appreciate your insights on this and this is clearly acknowledged as a limitation of this study. We assume that assessing the quality and accessibility of available screening services could be helpful in understanding the observed screening patterns.

2-Did cultural or religious practice affect the practice of screening for breast cancer seeing the rather low number (13%) in relation to an 87.7% awareness level of cancer. These were not explored in the study. It will be helpful to give more information on factors that could influence your results such as cultural beliefs, healthcare infrastructure, and accessibility.

**Response**: You are right we could account for additional potential confounders and even effect modifiers by including variables such as religion, cultural beliefs, healthcare infrastructure and social support systems. These could be future research directions for research in this domain of women’s health. Thank you for bringing this to our attention.

3-study design and setting – what health facility is located in this study areas, how accessible are they to study subjects and what infrastructure is on ground to help with breast cancer detection? Providing detailed information on the healthcare infrastructure or specific barriers faced by women in accessing these services will be helpful in appreciating the magnitude of the problem to readers.

**Response**: We have now revised the study design and setting section as suggested. Thank you.

Minor issues;
1. What informed the choice of rural women for this study seeing similar study had been done in female undergraduate students in the past?

**Response:** Our decision was informed by several critical factors. While previous studies among female university students provided valuable insights into breast cancer awareness in an educated, urban population, rural women (constituting approximately 55- 60% of The Gambia's female population) face unique challenges including geographical isolation, limited healthcare infrastructure, and financial constraints. Moreover, rural women often present with late-stage breast cancer due to delayed healthcare seeking, making them a crucial yet understudied population. Understanding their screening practices and barriers is essential for developing targeted interventions and informing health policies aimed at reducing rural-urban health disparities. Therefore, this study complements, rather than duplicates, previous research by addressing a critical knowledge gap in breast cancer screening practices among a vulnerable and underserved population.

2. Strengths and limitations (page 11) – although the population is nearly homogeneous for example proximity to health care facility with screening services could affect their screening practices regarding breast cancer.

**Response**: Thank you for the insights and we have now expanded on our study limitation as expected.

**Reviewer #2:**

Topic: Exploring Breast Cancer Awareness and Screening Practices amongst Gambian Women in the Gambia; Community-based- cross-sectional study

Dear Editor PLos One Journal,
Many thanks for the opportunity to review this paper from the Gambia. I am grateful.
General Comments
A good and interesting paper. It adds to the breast cancer literature from the Gambia. However, the topic needs revision.
The background outlined the key issues related to breast cancer awareness and screening practices in the Gambia
The statistical analysis were appropriate
The findings presented were relevant and the discussion gave an in-dept explanation on the topic.
Conclusion: the conclusion is generally sound and based on the findings; however, it needs a slight revision.

**Response**: We appreciate your insightful comment, Thank you.

Specific Comments
1. Topic: One of the study variables looked at nationality: Gambian and non-Gambian. Therefore, the authors should consider removing the term Gambian on the topic. The proposed topic should read, "Exploring Breast Cancer Awareness and Screening Practices amongst Women in the Gambia: A Community-Based cross-sectional Study.”

**Response**: We now updated the title as suggested and we have also improved our study’s eligibility criteria to adequately address our inclusion and exclusion criteria.

2. What was the justification to choose simple random sampling to select participants in households with more than a single eligible respondent on line 130-131? Secondly, how were/were households with no eligible respondent dealt with in the study?

**Response:** Simple random sampling was chosen for selecting participants within households with multiple eligible respondents to ensure equal probability of selection and minimize selection bias that could arise from convenience sampling or voluntary participation. This method helped prevent overrepresentation of more available household members (e.g., unemployed or housewives) who might have different screening behaviors. Regarding households without eligible respondents, these were documented and factored into our response rate calculations to maintain methodological transparency, with replacements systematically selected from the next household in a predetermined direction until we achieved our target sample size. We have reflected these strategies in the manuscript as suggested. Thank you.

3. Were the research assistant from Solace foundation trained for the data collection at the community level?

**Response:** Yes, all research assistants underwent standardized training conducted by the Solace Foundation, which included instruction on data collection using the Kobo Toolbox, interviewing techniques, and translation protocols for three major local languages (Mandinka, Wolof, and Fula). Prior to field deployment, the trained research assistants participated in a pilot study in the West Coast Region to ensure proficiency in data collection procedures and consistency in questionnaire administration.

4. In Table 1 and table 2, authors were using participants and in other tables respondents. They should be consistent with the terms. Table 2 should read “Breast cancer awareness of respondents.”

**Response:** We now change all of them to respondents as suggested.

5. In lines 202 and 207, the term “our respondents” should be changed to “the respondents” all throughout the document.

**Response:** We now change all of them to respondents as suggested.

6. Table 2: all variables with multiple respondents should be indicated, eg “ Knew the preventive methods of breast cancer risk.”

**Response:** This is implemented as suggested. Thank you.

7.Line 239 Kinteh et al , no year was added to the reference. Similar to Sanyang et al., line 290. Therefore, it should be rephrased

**Response:** We provide the year and rephrased accordingly. Thank you.

8. Conclusion Line 330-332: “The predictors of breast cancer screening practices were influenced by the parity of the women and the current occupation of the respondents” . Do they mean ethnicity? Parity was not, even though some of its sub categories indicated so.

**Response:** Thank you for this important clarification. Our conclusion correctly identifies occupation and parity categories (specifically women with two, three, and five children) as significant predictors of breast cancer screening practices, not ethnicity. While ethnicity showed some variations in screening uptake, none were statistically significant (p>0.05). Thank you.

Reviewer #3:

The article provides interesting findings on breast cancer in The Gambia. I think the paper is worthy of publication, but I have a few questions and suggestions for improvement with regard to the methodology and the evaluation strategy.

Response: Thank you for those comments.

The authors should better justify the statistical model they chose with mathematical expressions for the models. The descriptive results are not sufficient, charts can be used to support the results.

**Response:** We appreciate your suggestion for enhanced statistical presentation. We have now included a detailed mathematical expression of our binary logistic regression model, where logit(p) = ln(p/1-p) = β₀ + β₁X₁ + β₂X₂ + ... + βₖXₖ, with *p* representing the probability of breast cancer screening practice and *β* coefficients representing the effects of our predictor variables. This model was chosen due to the binary nature of our outcome variable (screening: yes/no) and its ability to estimate adjusted odds ratios while controlling for multiple predictors simultaneously. To enhance visual interpretation of our results, we have also added a bar chart depicting breast cancer screening practices distribution and another visualizing the main barriers to screening, which complement our tabulated findings and provide a clearer representation of the patterns observed in our data.

Although, the article demonstrated scholarly argument, the article is poorly written with a lot of typos and grammatical errors. The paper needs some editing and proof-reading as well as some double-check for consistency in spelling ( I have indicated some out of so many).
Accept with major revision

**Response:** We now revised the manuscript and addressed typos and grammatical errors accordingly. Thank you.

Reviewer #4:

General comment
The study aimed to explore breast cancer awareness and screening practices of rural Gambian women. This is titled “Exploring Breast Cancer Awareness and Screening Practices 1 Amongst Women in The Gambia; Community-based- cross-sectional study”. The study is premised on the limited strategies for addressing breast cancer in The Gambia.

**Response:** Thank you for the comment.

Major comment
Research on breast is laudable. However, the more specific and focused research is, the better. The study focused is inadequately articulated and contextualised. Absence of the operational definition of the main outcome variable of interest is a concern. Nonetheless, I suggest the authors focus solely on breast cancer screening practices.

**Response:** We have carefully revised the manuscript to sharpen its focus and context, specifically examining both breast cancer awareness and screening practices among rural women in The Gambia. The dual focus is intentional and necessary, as understanding awareness levels provides crucial context for interpreting screening behaviors, particularly in rural settings where health literacy and healthcare access intersect. We have now included clear operational definitions of our outcome variables, with breast cancer screening practice defined as "ever having undergone any form of breast cancer screening (breast self-examination, clinical breast examination, or mammography)." Throughout the manuscript, we have maintained consistent attention to both awareness and screening practices, as these are inextricably linked in the Gambian rural context, where understanding the relationship between knowledge and preventive health behaviors is crucial for developing effective interventions. This approach allows for a comprehensive understanding of the barriers to screening uptake while maintaining a focused analysis of screening practices as our primary outcome of interest.

Specific comments
Title:
• I suggest modification of the title to read as “Barriers and associated characteristics of breast Cancer Screening Practices amongst women in northern part of The Gambia;”
**Response:** We appreciate your suggested title modification. However, we respectfully prefer to maintain our current title "Exploring Breast Cancer Awareness and Screening Practices Amongst Rural Women in The Gambia: Community-based Cross-sectional Study" as it more accurately reflects our study's comprehensive scope, methodology, and findings as suggested by reviewer 2. While barriers and associated characteristics are important components of our analysis, our study examined both awareness and screening practices as interconnected aspects of breast cancer prevention in rural communities. Additionally, our current title explicitly identifies the study design and target population, providing immediate context for readers, and aligns with standard epidemiological reporting guidelines for observational studies.

Abstract:
• Background
o Lines 20-21: could be replaced with justification relating to breast cancer screening practices
o Line 22: study objective may thereafter reflect the modified title.

**Response:** We have now revised our abstract as suggested. Thank you

• Methods: consider recasting the statement to reflect the following
o Line 25: Why among rural women?? This is not reflected in the body of the work
o Lines 26-27: authors should recast … interviews may be conducted using structured questionnaires deploy using Kobo toolboxo Line 28: delete the phrase “SPSS version 28” - Avoid inclusion of software in abstract.

**Response:** Implemented as suggested in our revised version. Thank you
o Line 29: … to determine correlation?? Pearson Chi-square is used to examine association between two categorical variables
o Line 30: replace test with model
o Provide a statement to indicate that adjusted odds ratios was reported in the Results section
**Response:** We have revised and addressed our abstract’s method section accordingly. Thank you.

• Results:
o Line 34: replace the pronoun “our” with “the”, avoid the use of such pronoun as much as possible
o Lines 36-37: I could not find the result in the body of the work to justified the inclusion of the statement as “risk factors”. However, I guess the authors meant to say respondents’ characteristics
o Lines 39-42: please improve on the reporting style for the statement as only occupation and parity are reported.

**Response:** Implemented as suggested in our revised version. Thank you

• Conclusions:
o Line 43: average of what??? The statement is unclear!
o Line 44: what makes it optimal ... this is not indicated in the result nor in the method section
o Line 45: ... could not be traced to any of the result presented in the Results section of the abstract. Nonetheless, if the suggested title is sustained, it could be appropriate provided that it is linked to the results section.

**Response:** We have revised our conclusion and all your concerns are adequately addressed. Thank you

Introduction:
o Lins 58-59: the statement requires appropriate referencing
o Lines 83-86: the two statements are contradictory; first, lack of knowledge and second, good knowledge
o Line 99 as commented earlier – operational definition of the main outcome of interest is required
o The study justification is rather weak with respect to with the BC awareness or its screening practice among the target population, Most importantly, non-inclusion of the targeted population known practices towards BC screening
**Response:** We appreciate these valuable comments and have addressed them in our revised introduction. We have added appropriate references to support the statement about breast cancer diagnosis and mortality rates in The Gambia. We've clarified the apparent contradiction regarding knowledge levels by explaining that while university students showed good awareness, this finding may not represent rural women who face different challenges. We've now included a clear operational definition of breast cancer screening practices as "ever having undergone any screening method (breast self-examination, clinical breast examination, or mammography)." The justification has been strengthened by emphasizing the knowledge gap regarding rural women's screening practices and the urgent need to understand barriers in this understudied population. Thank you.

Methods:
• Study design and setting
o Lines 107-111: Authors should mention no of districts per the Local government authorities in the northern part of the Gambia. This should be followed by the average numbers of communities per district. Expectedly, the setting should also include the spread of the screening centres
**Response:** Thank you for this constructive feedback. We have enhanced the study setting description to provide more comprehensive geographical and healthcare infrastructure details. The Northern Gambia comprises two local government authorities: Kerewan Area Council with four districts (Upper Baddibu, Lower Baddibu, Central Baddibu, and Jokadu) and Kuntaur Area Council with three districts (Upper Saloum, Lower Saloum, and Niani). Each district contains an average of 25-30 communities. Regarding screening infrastructure, the region has one district hospital in Kerewan offering clinical breast examination, six major health centers (four in Kerewan and two in Kuntaur), and twenty-eight community health posts. However, mammography services are only available at Edward Francis Small Teaching Hospital in Banjul, approximately 200 kilometers from most study communities.

• Sample size determination
o Line 120: the statement “confidence interval of 99% with 50% precision” is unclear! Cross sectional one population study estimated sample size usually utilised within 1 – 5% level of precision. Authors are advised to state the correct parameters used to arrive at 985 as the estimated sample size.

**Response:** We appreciate your observation about the sample size calculation parameters. We have clarified this in our revised manuscript to accurately reflect that we used a 5% precision (not 50%) at a 99% confidence level, assuming 50% prevalence of breast cancer awareness since no previous community-based studies were available in our setting. Using the formula n = [Z²₁₋ₐ/₂ × P(1-P)]/d², where Z₁₋ₐ/₂ is 2.576 (99% confidence level), P is 0.5 (50% expected proportion), and d is 0.05 (5% precision), we calculated a minimum sample size of 662. After accounting for a 10% non-response rate and design effect of 1.5 due to our multi-stage sampling technique, our final target sample size was 1,092, from which we achieved 985 completed responses (97.3% response rate).

• Sampling strategy
o Lines 123-125: This is not a selection stage.
o Lines 125-131: Questions that ought to have been answered by the “setting” section
♣ Districts selected - out of how any districts?? Total districts in each LGAs should have been mentioned under the study setting
♣ what is the average community per district??
♣ How many households selected per community???
♣ how many participants selected per household ... where more than one was eligible??
**Response:** Thank you for these detailed observations about our sampling strategy description. We have addressed these points in our revised study setting section, which now clearly specifies that Kerewan Area Council has four districts and Kuntaur Area Council has three districts, with each district containing an average of 20-30 communities. Regarding our sampling process, we employed probability proportional to size to determine household allocation per community, with an average of 25-30 households selected per community. In households with multiple eligible respondents, only one participant was randomly selected using a random number generator to ensure unbiased selection. This systematic approach to sampling helped ensure representative coverage of our target population while maintaining methodological rigor in participant selection.

• Data collection and management
o Line 132: replace the sub-section title with “Data collection instruments”
o Line 150: it may be necessary to provide justification for using 3 local languages
**Response:** Thank you for these suggestions. We have revised the section title as recommended and have clarified our language selection rationale. Mandinka, Wolof, and Fula were chosen as they are the predominant languages in our study area, collectively spoken by approximately 92% of the population in Northern Gambia, thus ensuring effective communication with most participants while maintaining feasible training and quality control processes.

• Authors are advised to insert a subsection titled “Study variables”. This should clearly state the operational definition of the outcome variable and the selected characteristics of the women.

Response: We have provided a subsection in the revised manuscript as suggested. Thank you

• Statistical analysis
o Line 158: replace the sub-section title with “Data management and analysis”
o Lines 164-165: replace with “All analysis was conducted at a 5% level of significance”
Results:
• insert “Women’s characteristics” as a subsection
• Line 175: How figure (1012) was obtained needs to be clearly stated in the Methods - sampling strategy subsection

**Response:** We have addressed this in the revised manuscript accordingly.

• Association of the selected women’s characteristics and breast cancer screening
o Line Technically, the statement “In total, 529 households were included in the study, with 273 children aged 12-59 months enrolled” could be not right. More than a child could come from a household but NOT the other way round.
o Again, there is no table/figure that is referenced

**Response:** We have addressed this in the revised manuscript accordingly. We have clearly referred figures 1 and 2 wherein required as suggested. Thank you.

• Table 1
o While combining the distribution of women by their characteristics and the characterises association with breast cancer is welcome, I will suggest that

**Response:** Thank you for your comment. We combine univariate and bivariate to enhance readability and maximize the number of tables to be used in this paper.

o 1. Total column should come before the columns for Yes and No for breast cancer screening
o 2. Yes and No columns of breast cancer screening percentages should be presented in row-wise”.

Response: Thank you for your thoughtful suggestions regarding table organization. While we acknowledge that presenting row percentages can be useful for comparing screening rates within categories, we deliberately chose column percentages to highlight the distribution of characteristics among screeners versus non-screeners, which aligns with our primary research objective of understanding the profile of women who undergo screening. However, we also place the total column after our two binary columns to conform with the contemporary presentation of chi-square results based on your research questions as it would improve readability. Since chi-square mathematically measures group-level differences between our observed and expected values, you can present your column or row totals based on your research question for contextuality and interpretability. Thank you.

o The use of chi-square becomes inappropriate in situations where there is zero value in any of the contingency table cells

**Response:** We have not provided an “a” notation to represent Ficher’s Exact Test that was used where the specific stratum-level expected values are less than 5. We have also updated both the abstract and analysis section to reflect these updates. Thank you.
o Authors should ensure all the variables listed in Table 1 are clearly defined under the methods, study variables subsection

**Response:** We now provided “Study variable” subsection in the method section to clearly describe the study variables and how they are used in this paper.

o Table 2 – should appear before the presentation of the “Association between the selected women characteristics and the breast cancer screening practice
**Response:** This is revised as suggested. Thank you.

• Discussion
• The unclear focus of the study, lines 234-237 in particular, is a concern here. Nonetheless, authors are advised to avoid writing the results under the discussion section – see lines 302 – 317

Response: We have revised these sections accordingly. Thank you.
