# Supplementary material for: Exploring breast cancer awareness and screening practices amongst rural women in The Gambia: Community-based cross-sectional study
Source: PLoS One. 2025 May 12;20(5):e0307785. doi: 10.1371/journal.pone.0307785 (PMC12068578; doi:10.1371/journal.pone.0307785)
Supplement: Attachment — Submitted filename: Response_to_reviewers_auresp_2.docx [file pone.0307785.s008.docx]

January 8, 2025

Manuscript ID: [PONE-D-24-19264] - [EMID:b560eba95e797023]

**Title:** “Exploring Breast Cancer Awareness and Screening Practices Amongst Rural Women in The Gambia: Community-based Cross-sectional Study”

RE: Revised manuscript submission and response to reviewers’ comments

Dear Editor,

This letter is in reference to your email dated January 2, 2025 with reviewers’ comments. We are very pleased that the manuscript is potentially acceptable for publication in *PLOS ONE* once we have carried out the revisions.

We would like to thank the reviewers for these insightful and helpful comments and for giving us the chance to revise our manuscript. We believe the revised manuscript has been significantly improved and the reviewers’ comments have been addressed adequately. We think in its current form it will make a valuable contribution to the literature on this increasingly important topic. 

Please find for your kind consideration the following:

- A section-by-section response to the comments and suggestions of the reviewers (below).
- The revised manuscript, provided as a marked-up copy and a clean copy.

We hope that these changes meet with your favourable consideration. Please do not hesitate to get in touch if you require any further information. 

Bakary Kinteh
Corresponding Author 

Reviewer #1:

**General comment** The manuscript has improved considerably. However, I still a bit confused on the main outcome of interest operational definition. To my understanding, the terms "breast cancer screening practices" and "breast cancer screening uptake" refer to different aspects of breast cancer screening. While screening practices are about the methods and guidelines for conducting screenings, uptake relates to how many people actually get screened. Nonetheless, exploring the two concepts is essential at improving breast cancer prevention efforts and achieving better health outcomes. I therefore advise that the two concepts be clearly delineated in the body of the manuscript, as clearly stated in the objective of the study (Lines 24-26).

**Response**: Thank you for pointing this out. The manuscript has been revised to clearly define "breast cancer screening uptake" as the primary outcome of interest. Operational definitions of both "screening uptake" and "screening practices" have been clarified, and references to "screening practices" have been replaced with "screening uptake" where appropriate. The study objectives have also been updated to reflect this focus.

**Specific comments**

Title: • If word the “Northern” will not appear on the title, I suggest how the region was selected be clearly mentioned under the sampling strategy subsection of the Method section.

**Response**: Thank you for this observation. The manuscript has been updated to include a clear explanation of the rationale for selecting the northern region in the Sampling Strategy subsection. The region was purposively chosen due to its high population of underserved rural women and limited healthcare access, making it a representative setting for the study's objectives.

Abstract:

• Methods:
o Line 28: I suggest the word Northern be deleted if it will not be retained in the title as commented above

**Response**: Revised and deleted as suggested.
o Line 31: As explained under the general statement, I suggest the phrase “breast
cancer screening practices” be replaced with “**breast cancer screening uptake**”

**Response**: Revised and replaced as suggested.
o Lines 32-33: authors should recast …
o Provide a statement to indicate that adjusted odds ratios was reported in the Results section

**Response**: Revised as suggested.

• Results:
o aOR – adjusted Odds Ratio is mentioned without being defined at first use. This could be avoided if the comment above is addressed.

**Response**: This is revised and provided as suggested. Thank you.

• Conclusions:
o Line 45: what makes it suboptimal ... the use of the statement is unclear
o Lines 45-46: The statement “The key barriers include knowledge deficits, service unavailability, and cost constraints” could not be linked with any of the result presented in the Results section of the abstract.

**Response:** We appreciate your feedback. The abstract has been revised to directly reference the identified barriers from the Results section, including a lack of knowledge (58.7%), service unavailability (13.5%), and financial constraints (13.1%), ensuring consistency between the abstract and the results.

Introduction:
o Lines 85-86: Authors should recast the objective statement to include the screening uptake

**Response**: This is revised as suggested. Thank you.

Methods:

• **Study design and setting**
o Lines 94: the phrase … northern region of … should be addressed as commented earlier
o Line 105: I suggest the deletion of the phrase “Healthcare Access and Barriers”
o Lines 105-115: There is a need to reference some of the factual statements in the paragraph

**Response**: We revised and addressed accordingly. Thank you.

• **Study population and eligibility criteria**
o Lines 120-125: Authors are advised to delete the exclusion statement in lines 123- 124, as exclusion criteria statement is NOT expected to be opposite of inclusion criteria statement. Expectedly, exclusion criteria statement should addressed only women that would not be eligible out of the women already included in the study

**Response**: We removed as suggested. Thank you.

**Study variables**
o Line 127: As mentioned earlier, the main outcome variable was the “breast cancer
screening uptake”. This should be reflected in other sections of the study. The evidence is clear from the analysis.

**Response:** We revised and addressed accordingly. Thank you.

**Sampling strategy**
o Lines 152-166: The first two stages, as mentioned here, are not selection stages. The selected women were from the two LGAs with a total of seven districts, in the northern region of The Gambia.

**Response:** We revised and addressed accordingly. Thank you.

o As I explained earlier, if Northern will not be included in the title, I advise the selection of the Northern region be the first selection stage. Is it selected purposively or by any other method?
o If the word northern is included in the title, the 3 rd stage should become the 1 st stage, in that order.

**Response:** We revised and addressed accordingly. Thank you.

**Data management and statistical analysis**
o Lines 194-196: replace the statement with “Descriptive statistics such as means with standard deviations (SD) for continuous variables, and frequencies and percentages for categorical variables were used present the women characteristics including their breast cancer awareness and screening practices”.
o Line 198: replace “practices” with “uptake”

**Response:** We revised and addressed accordingly. Thank you.

o Line 225: The clause “and statistical significance was declared at p<0.05.” may be deleted as the statement is already implied by the use of 95% confidence interval.

**Response:** Thank you for your observation. We acknowledge that the use of 95% confidence intervals implies statistical significance. However, we have retained the reference to *p<0.05* as a separate sentence to ensure clarity, particularly for bivariate analyses (e.g., chi-square and Fisher's exact tests), where statistical significance is explicitly assessed using *p-values*. This addition ensures consistency and reinforces the approach used in both bivariate and multivariable analyses.

Results: • Line 237: Sociodemographic characteristics … of what??? I suggest “Women’s sociodemographic characteristics” as the subsection title • Lines 238-239: The statement “Of the 1,012 women recruited from thirty-five communities, 985 completed the study (response rate: 97.3%).” is not a result. Authors should delete the statement. Besides, it is clearly mentioned under the Methods section.

**Response:** We revised and addressed accordingly. Thank you.

• Figure 1: delete the figure, but ensure a total row (last row) is created in Table 1

Response: Thank you for your suggestion. We acknowledge the importance of presenting data in a concise and meaningful way. However, we believe that retaining Figure 1 is crucial for visualizing the distribution of breast cancer screening uptake, which is a key finding of this study. Table 1 already provides detailed sociodemographic characteristics cross-tabulated by screening uptake, while Figure 1 serves to graphically represent the overall proportion of participants who underwent screening. Removing Figure 1 would compromise the clarity and emphasis on this critical finding. We propose keeping Figure 1 and ensuring Table 1 maintains its focus on sociodemographic determinants of screening uptake without introducing redundancy.

• Line 251: replace the subsection with “Association between the selected women’s
characteristics and breast cancer screening uptake”,

**Response:** We revised and addressed accordingly. Thank you.

o Importantly, this should be presented after the beast cancer awareness and practices descriptive presentations. • Table 1

**Response:** We appreciate your perspective on the order of results presentation. However, we believe that retaining the current arrangement, with Table 1 appearing first, is appropriate. Table 1 presents the sociodemographic characteristics of the respondents, which is foundational information that provides essential context for interpreting the subsequent findings on breast cancer awareness and screening uptake. Introducing the sociodemographic data first ensures a logical flow, helping readers better understand the population being studied before delving into awareness and practices. We believe this arrangement enhances the clarity and coherence of the results section, and we kindly request to maintain it as is. Thank you for your understanding.

o I quite understand that arrangement of table’s columns, including rowwise/column-wise percentages presentation, does not influence the chi-square result. However, for sake of clarity to the potential Plos One reader, my previous suggestion may be necessary, especially in relation to the statement in lines 256- 258.

**Response:** This is implemented as suggested. Thank you.

o Additionally, I suggest that the title should read as “Distribution of women’s sociodemographic characteristics by breast cancer screening uptake

**Response:** This is implemented as suggested. Thank you.

• Lines 265-275: The paragraph should have a subsection title “**Breast cancer**
**awareness**”

**Response:** This is implemented as suggested. Thank you.

• Lines 279-280: delete the statement on breast cancer screening uptake. As suggested earlier, Table 1 should be sufficient to indicate it.

**Response:** This is implemented as suggested. Thank you.

• Figure 2: should be renamed Figure 1

**Response:** We still maintained Figure 1 and 2 as initially assigned but we have reformatted Figure 2 as suggested. Thank you.

o It will be nice if the figure is reproduced in descending order of percentage magnitudes
o Its title may be replaced with “Distribution of breast cancer’s signs and symptoms”

**Response:** This is implemented as suggested. Thank you.

o It may be placed as an appendix • Table 4 – correct as Table 3

**Response:** We now correctly renamed Table 3 as suggested. Thank you.

o Its title may be replaced with “Distribution of breast cancer screening practices”

**Response:** This is implemented as suggested. Thank you.

o Delete first items “Have you ever practiced any breast cancer screening?”, to avoid repletion of information

**Response:** Deleted as suggested. Thank you.

• Line 301: replace with “**Predictors of breast cancer screening uptake**”

**Response:** This is implemented as suggested. Thank you.

• Table 4
o Replace the title with “Adjusted Odds Ratios of breast cancer screening uptake
by women’s selected characteristics”

**Response:** This is implemented as suggested. Thank you.

• **Discussion**
o Line 335: breast cancer knowledge??? A composite score would be needed to speak to this, which is not included in the work. Of course, the focus is not on knowledge; authors are enjoined to avoid the mix-up between awareness and knowledge.

**Response:** Thank you for your insightful comment. We acknowledge the distinction between awareness and knowledge. The focus of this study is on breast cancer awareness, as defined by participants’ familiarity with the term “breast cancer” and recognition of its signs, symptoms, and general risk factors.

To address this, we have replaced references to "knowledge" with "awareness" throughout the manuscript, particularly in Line 335. This ensures clarity and alignment with the study’s focus.
