# Supplementary material for: Exploring breast cancer awareness and screening practices amongst rural women in The Gambia: Community-based cross-sectional study
Source: PLoS One. 2025 May 12;20(5):e0307785. doi: 10.1371/journal.pone.0307785 (PMC12068578; doi:10.1371/journal.pone.0307785)
Supplement: Attachment — Submitted filename: Response to reviewers_2025.docx [file pone.0307785.s010.docx]

January 20, 2025

Manuscript ID: [PONE-D-24-19264R2] - [EMID:6ca1f9d03a4dfd20]

**Title:** “Exploring Breast Cancer Awareness and Screening Practices Amongst Rural Women in The Gambia: Community-based Cross-sectional Study”

RE: Revised manuscript submission and response to reviewers’ comments

Dear Editor,

This letter is in reference to your email dated January 2, 2025 with reviewers’ comments. We are very pleased that the manuscript is potentially acceptable for publication in *PLOS ONE* once we have carried out the revisions.

We would like to thank the reviewers for these insightful and helpful comments and for giving us the chance to revise our manuscript again. We believe the revised manuscript has been significantly improved and the reviewers’ comments have been addressed adequately. We think in its current form it will make a valuable contribution to the literature on this increasingly important topic. 

Please find for your kind consideration the following:

- An article published by Ramya Ahmad et al, 2019 is a retracted paper and removed from our citation and references list.
- A section-by-section response to the comments and suggestions of the reviewers (below).
- The revised manuscript, provided as a marked-up copy and a clean copy.

We hope that these changes meet with your favourable consideration. Please do not hesitate to get in touch if you require any further information. 

Bakary Kinteh
Corresponding Author 

Reviewer #1:

**General comment** The manuscript has improved considerably. However, I still a bit confused on the main outcome of interest operational definition. To my understanding, the terms "breast cancer screening practices" and "breast cancer screening uptake" refer to different aspects of breast cancer screening. While screening practices are about the methods and guidelines for conducting screenings, uptake relates to how many people actually get screened. Nonetheless, exploring the two concepts is essential at improving breast cancer prevention efforts and achieving better health outcomes. I therefore advise that the two concepts be clearly delineated in the body of the manuscript, as clearly stated in the objective of the study (Lines 24-26).

**Response**: Thank you for pointing this out. The manuscript has been revised to clearly define "breast cancer screening uptake" as the primary outcome of interest. Operational definitions of both "screening uptake" and "screening practices" have been clarified, and references to "screening practices" have been replaced with "screening uptake" where appropriate. The study objectives have also been updated to reflect this focus.

**Specific comments**

Title: • If word the “Northern” will not appear on the title, I suggest how the region was selected be clearly mentioned under the sampling strategy subsection of the Method section.

**Response**: Thank you for this observation. The manuscript has been updated to include a clear explanation of the rationale for selecting the northern region in the Sampling Strategy subsection. The region was purposively chosen due to its high population of underserved rural women and limited healthcare access, making it a representative setting for the study's objectives.

Abstract:

• Methods:
o Lines 21-23: replaced with the statement “This study aimed to assess breast cancer awareness and identify associated factors influencing screening uptake among rural women in The Gambia”

**Response**: Revised and replaced as suggested.

Methods:

o Line 25: replace the word “Gambia” with “The Gambia”, and elsewhere in the manuscript

**Response**: The word Gambia is revised and replaced as suggested (The Gambia) throughout the manuscript.
o Line 29: replace the phrase “Chi-squared and/or Fisher’s exact test” with “Chisquared or Fisher’s exact test, as appropriate”

**Response**: Revised as suggested.

o Line 29: replace the phrase “were reported was in the results.” with “were reported”

**Response**: Revised as suggested.

• Results:
o Line 36: replace the phrase “Multivariable analysis revealed that students” with “Students”.

**Response**: This is revised as suggested “Students”. Thank you.

• Conclusions:
o Lines 43-44: Authors are advised to include result(s) suggesting the phrase “primarily due to limited knowledge of the urgent need for accessible and affordable screening services” in the Results section of the abstract

**Response:** We appreciate your feedback. The conclusion has been revised to directly reference the identified barriers from the Results section, including a lack of knowledge (58.7%), service unavailability (13.5%), and financial constraints (13.1%), ensuring consistency between the conclusion and the results.

o Lines 44-45: Let the recommendation statement be presented in a separate sentence.

**Response:** The recommendation statement is presented in a separate sentence as suggested.

Methods:

• **Study design and setting**
o Lines 94: the phrase … northern region of … should be addressed as commented earlier
o Line 105: I suggest the deletion of the phrase “Healthcare Access and Barriers”
o Lines 105-115: There is a need to reference some of the factual statements in the paragraph

**Response**: We revised and addressed accordingly. Thank you.

• **Study population and eligibility criteria**
o Line 119: replace with “Study population”

**Response**: We replaced as suggested. Thank you.

**Sample size determination**
o Lines 146-148: Authors should ensure the correctness of the following stated figures!

1. Estimated minimum sample size becomes 1103 (not 1092 as indicated) if adjusted for 10% non-response with 1.5 design effect

2. Besides, let’s assume 1092 is correct! Then, only of 1012 (not 1092) that 97.3% response rate would yield 985 completed the study

**Response:** We revised and addressed accordingly. Thank you.

**Sampling strategy**
o Lines 151-162: replace with “A four-stage sampling method was employed. First, the northern region was purposively selected due to its geographical location, high population density of underserved rural women, and absence of mammography services. This selection aligns with the study's aim of understanding screening uptake in rural, as it represents a significant proportion of rural communities with limited healthcare access. The northern region of The Gambia comprises of Kerewan and Kuntaur local government areas (LGAs), with four (Upper Baddibu, Lower Baddibu, Central Baddibu, and Jokadu) and three (Upper Saloum, Lower Saloum, and Niani) districts, respectively. Second, five communities were randomly selected from each district, yielding 35 study communities in total. Third, within each selected community, households were sampled using probability proportional to size, selecting an average of 25-35 households per community. Finally, one eligible woman was selected from each household.”

o Also important to note, selection of 25-30 households in each of the 35 communities would not yield the minimum sample size estimated (30*35=1050; this cannot lead to 1092 assuming its correct). However, 25-35 households per community will do!

**Response:** We replaced and addressed accordingly. Thank you.

**Data management and statistical analysis**
o Lines 197-199: The women age was not covered with the present statement. I refer the authors to my previous advice to replace the statement with “Descriptive statistics such as means with standard deviations (SD) for continuous variables, and frequencies and percentages for categorical variables were used present the women characteristics including their breast cancer awareness and screening practices”. o Line 199: replace “We employed Pearson's chi-squared and/or Fisher’s exact test to” with “As appropriate, Pearson's chi-squared or Fisher’s exact test was used to … ” o Lines 204-207: Authors are advised to place the statement “Despite its non-significance at the bivariate analysis level, ethnicity was retained in the model owing to its theoretical importance and our specific interest in understanding its effects on breast cancer screening uptake in relation to other predictors” immediate on line 211 after “bivariate level.” to facilitate a smooth narrative.

**Response:** We revised and addressed accordingly. Thank you.

Results:

• Figure 1:

o Line 251: replace “practices” with “uptake”

• Line 251: replace the subsection with “Association between the selected women’s
characteristics and breast cancer screening uptake”,

**Response:** We revised and addressed accordingly. Thank you.

• Table 1:

o For sake of clarity to the potential Plos One readers, my previous suggestion is necessary!

▪ Column 2 to be presented as Total (column percentage), while others remain as they were

▪ By so doing, statements on women’s characteristic in lines 240-247 could be referenced adequately.

**Response:** This is implemented as suggested. Thank you.

o Additionally, I suggest that the title should read as “Distribution of women’s sociodemographic characteristics by breast cancer screening uptake

**Response:** This is implemented as suggested. Thank you.

Lines 291: I suggest the paragraph subsection title be read as **“Breast cancer screening practices”** as this includes BC signs and symptoms.

**Response:** This is implemented as suggested. Thank you.

• Lines 299-302: The statement “These findings highlight the complex interplay of structural, knowledge-based, and personal barriers influencing breast cancer screening uptake in this rural population, suggesting the need for targeted interventions to address these multiple barriers to screening.” should be moved under Discussion section

**Response:** This is implemented as suggested. Thank you.
